# Sol-Gel Entrapped Levonorgestrel Antibodies: Activity and Structural Changes as a Function of Different Polymer Formats

**DOI:** 10.3390/ma4030469

**Published:** 2011-02-25

**Authors:** Moran Shalev, Altstein Miriam

**Affiliations:** Department of Entomology, Institute of Plant Protection, The Volcani Center, Bet Dagan 50250, Israel; E-Mail: smorans@tx.technion.ac.il

**Keywords:** immunoaffinity purification, sol-gel, antibodies, levonorgestrel, TMOS, THEOS, polyethylene glycol

## Abstract

The paper describes development of a sol-gel based immunoaffinity method for the steroid hormone levonorgestrel (LNG) and the effects of changes in the sol-gel matrix format on the activity of the entrapped antibodies (Abs) and on matrix structure. The best sol-gel format for Ab entrapment was found to be a tetramethoxysilane (TMOS) based matrix at a TMOS:water ratio of 1:8, containing 10% polyethylene glycol (PEG) of MW 0.4 kDa. Addition of higher percentages of PEG or a higher MW PEG did not improve activity. No activity was obtained with a TMOS:water ratio of 1:12, most likely because of the very dense polymer that resulted from these polymerization conditions. Only minor differences in the non-specific binding were obtained with the various formats. TMOS was found to be more effective than tetrakis (2-hydroxyethyl)orthosilicate (THEOS) for entrapment of anti-levonorgestrel (LNG) Abs. However, aging the THEOS-based sol-gel for a few weeks at 4 °C stabilized the entrapped Abs and increased its binding capacity. Confocal fluorescent microscopy with fluorescein isothiocyanate (FITC) labeled immunoglobulines (IgGs) entrapped in the sol-gel matrix showed that the entrapped Abs were distributed homogenously within the gel. Scanning electron microscopy (SEM) images have shown the diverse structures of the various sol-gel formats and precursors.

## 1. Introduction 

Sol-gels are composites formed by a chemical process in which metallic or semi-metallic alkoxide precursors or their derivatives undergo a chemical reaction that involves hydrolysis followed by condensation and polymerization. Most sol-gels are silicon-based oxides, although they may also be based on other compounds, such as aluminum silicates, titanium dioxide, zirconium dioxide and many other oxide compositions. Silica-based (SiO_2_) sol-gel matrixes can be designed with a wide range of physical properties (e.g., porous texture, network structures, surface functionalities), and can be processed under a wide variety of conditions including ambient temperatures, moderate pH values and short gelation times, making silica alkoxides the most preferred precursors. The resulting matrixes may take the form of porous wet gels, ambigels, aerogels, xerogels, or organically modified sol-gels, (ormosils); they are characterized by a high surface area, controllable porosity, inertness and stability to chemical and physical factors, and exhibit optical clarity in the visible and ultraviolet ranges. Detailed reviews of the sol-gel process, the various sol-gel matrixes, and their properties have been published [1,2,3,4].

Since the important finding by Braun *et al*. [5], that enzymes can be entrapped in a silica-based matrix, many studies have addressed the entrapment of a wide variety of biological molecules, such as: enzymes; antibodies (Abs); regulatory proteins; transport proteins; membrane-bound proteins, including G-protein-coupled receptors (GPCRs); nucleic acids; liposomes; and even whole cells. The resulting matrices were implemented in a variety of applications (For review see [6,7,8,9,10,11] and references therein). In order to preserve the active conformation of the biomolecules within the sol-gel matrix, and to ensure optimal performance, it is necessary to optimize the configuration of the doped sol-gel with respect to the ranges of pH and ionic strength that are favorable for the biomolecules; to provide the pore size that ensures that the entrapped biomolecules gain access to substrates, ligands or analytes; and to minimize interactions between the sol-gel and the entrapped biomolecules. The past two decades have seen intensive studies of the nature and structure of sol-gels and their effects on the entrapped biomolecules, and these were discussed in detail in several reviews. Most of these studies, however, were carried out on enzymes or receptors, whereas only a few focused on Abs. The potentially wide scope of Ab-doped sol-gels in development of immunoaffinity purification (IAP) devices [12] highlights the need for further study with respect to sol-gel entrapped Abs.

Sol-gel biocomposites (*i.e.*, sol-gel matrices containing entrapped biomolecules) can be prepared from inorganic (silica or other metal or semi-metal) alkoxide precursors, or from combined organic/inorganic materials such as tetraethylorthosilicate (THEOS), tetramethylsilane (TMOS) or glycerated silanes (such as diglycerylsilane). Furthermore, additives can be encapsulated together with the entrapped biomolecules, in order to enhance their stability and activity. Such additives may be hydrophobic moieties, polymers (e.g., polyethylene glycol-PEG-glycerol, polyvinylimidazole, *etc*.), surfactants, liposomes, organic solvents (e.g., cyclohexane), polysaccharides (e.g., dextran, cellulose, and chitosan), cofactors (e.g., redox modifiers), or even biological or synthetic materials. These additives can be mixed directly with the sol-gel precursors before gelation, to form hybrid organic–inorganic gels; they may alter the physical properties of the gel, e.g., its rigidity, mechanical stability, pore size, and optical or electrochemical clarity, and may also affect the interactions of the gel with the entrapped biomolecules, so as to enhance the overall activity [13,14,15].

Although sol-gel-derived biocomposites have been shown to be useful in many analytical applications there are many unresolved issues which need further evaluation. For example, material properties still need to be improved in order to reduce cracking, age-linked shrinkage, and pore collapse; there is still ample scope for significant improvements in the physical and chemical parameters of precursors and of reaction conditions (e.g., the nature of the precursor and additives, hydrolysis ratios, presence of solvents and their nature, condensation kinetics, *etc.*); improved organic-inorganic composite materials are needed that might enhance the bioactivity of the entrapped molecules, and better understanding is needed of the protein-silica and analyte-silica interactions, which depend on electrostatic, hydrophilic, or hydrophobic interactions. Furthermore, it is still necessary to be able to optimize the physical properties of doped sol-gels with regard to size, shape, pore size, uniformity, *etc*., and thereby to enhance performance of the entrapped biomolecules in terms of reproducibility, sensitivity, and useful lifetime. 

In the present study, we tested some of the above parameters by using sol-gel-entrapped Abs generated against the steroid hormone levonorgestrel (LNG) [16], which belongs to the group of endocrine disruptors [17]. We examined the effects of changes in the sol-gel matrix format on the activity of the entrapped Ab and on matrix structure, as it relates to the properties of the porogen PEG at various molecular weights and concentrations, and under diverse hydrolysis conditions (*i.e.*, TMOS:H_2_O ratios). These studies examined Abs entrapped in gels in the form of monoliths generated from various precursors (TMOS *vs*. THEOS), and in aged gels. The results clearly revealed that the above parameters affected both the structure and activity of the monoliths, and strengthened the perception that optimization of biomolecule entrapment is needed in order to achieve maximal efficiency in any given application.

## 2. Results and Discussion 

Developments in sol-gel processing over the past few decades have demonstrated great potential and the advantages offered by this technology in many fields. The extension of this technology to the entrapment of functionally active proteins in silica made it even more attractive for demanding applications in bioorganic synthesis, medicine, biotechnology, and environmental technologies. Although this technique has been intensively explored, there is still a need to improve the chemical and physical parameters of the precursor and reaction conditions in order to improve the performance of the entrapped biomolecules and to resolve issues associated with cracking, aging, shrinking, and pore collapse of the casted matrix. 

Sol-gel-based IAP has gained a lot of attention in the past two decades and resulted in the development of many IAP devices. (For review see [3,6,7,12]). Despite the extensive use of this approach, only a few studies focused on the effects of the above parameters on the performance of sol-gel-entrapped Abs (for review see [12] and references therein) and even less studies addressed the structure-functions relationship of Ab doped sol-gel monoliths prepared under different conditions (*i.e.*, different combinations of precursors and porogens under various aging conditions). The present study explored the effects of differences in sol-gel formats on the activity of entrapped Abs and on the structure of the matrix, attempting to correlate between the activity of the entrapped Ab and the structure of the matrix. 

### 2.1. Effects of PEG on the Sol-Gel Structure and on the Activity of Entrapped Abs

**Figure 1:**
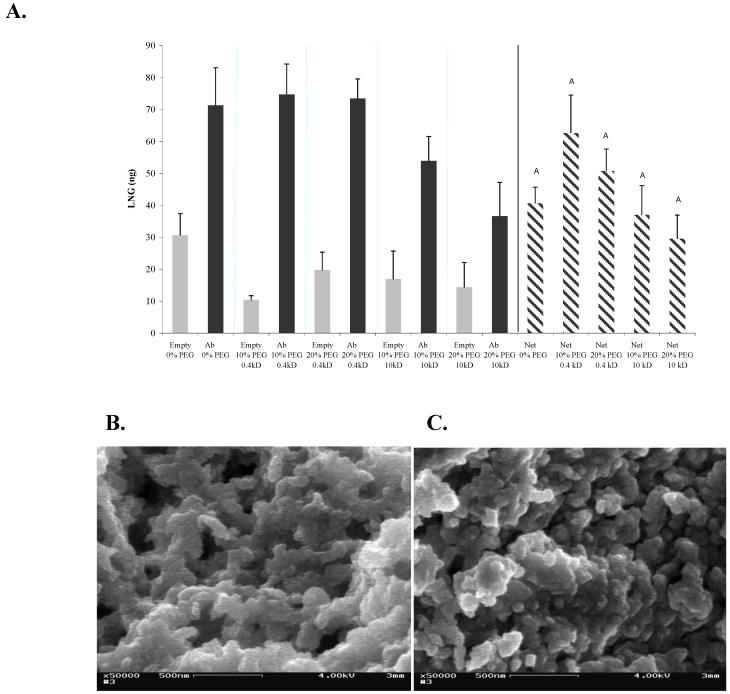
Effects of PEG on the structure and activity of entrapped Abs in 1:8-TMOS-based sol-gel. (**A**) LNG (100 ng) was applied on 1-day-aged sol-gel columns containing 0, 10 or 20% of 0.4-kDa or 10-kDa PEG. Amount of eluted LNG was determined by ELISA. Vertical bars labeled ‘Empty’ represent columns that were not doped with Abs, and therefore represent non-specific binding. Bars labeled ‘Ab’ represent the total binding capacity and bars labeled ‘Net’ represent the specific binding values (Ab minus empty). Each bar represents the mean ± S.E.M of n experiments, where: for 0% PEG, n = 2; for 10 or 20% of 0.4-kDa PEG, n = 6; for 10 or 20% of 10-kDa PEG, n = 4. Means with the same letter did not differ significantly at *p* < 0.05; (**B**) SEM image of 1:8 TMOS-based sol-gel containing 0% PEG; (**C**) SEM image of 1:8 TMOS-based sol-gel containing 10% of 0.4-kDa PEG. Magnification, ×50,000; scale bar represents 500 nm.

One of the major advantages of sol-gel-derived silicate materials in entrapment of proteins is the possibility of controlling their pore sizes during the preparation process. In order to be able to implement the technology for a variety of applications, it is necessary to form a matrix in which the pores are large enough to allow diffusion of small molecules into the matrix, but small enough to prevent diffusion of entrapped molecules out of the matrix. Thus, pore-size tuning is one of the most significant factors that enable the generic sol-gel technology to achieve entrapment of many kinds of biomolecules of diverse sizes for a wide range of applications. Alkoxy-silane-based sol-gel monoliths typically have pore-size distributions in the 4 to 200 nm range [18], therefore they cannot be used for certain applications, such as those that require entrapment of large molecules. In order to overcome this problem bimodal monoliths, which have two different ranges of pore sizes—nanometric and micrometric—have been developed over the years. In order to create such monoliths porogens were often used; these act both as through-pore templates and as solubilizers of the silane reagent. Water-soluble organic polymers such as PEG or polyethylene oxide (PEO) are the most frequently used porogenic agents in many sol-gel studies, and a direct correlation has been demonstrated between porogen MW and concentration on the one hand, and monolith structure, pore size and properties, on the other hand (for example see [19,20]). PEG and other porogens play several other important roles in sol-gel applications, apart from structure modification: they decrease the non-specific binding of proteins to the matrix by lowering the interaction of entrapped biomolecules with the monolith, and fill the pores and spaces in the matrix, thereby eventually preventing matrix shrinkage and collapse and so, in turn, protect the entrapped biomolecules from denaturation and activity loss (for review see 4, 13). Indeed, previous studies revealed that addition of PEG to sol-gel bio-composites significantly improved the activity of entrapped Abs and enzymes (for example see [21,22,23]); and Besanger *et al*. have shown that addition of 10-kDa PEG was more effective than that of 0.4-kDa PEG for encapsulation of various proteins, such as dopamine and acetyl-choline receptors. Our first goal within the present study was to determine the effect of PEG on the activity of anti-LNG Abs encapsulated within a sol-gel matrix. The anti-LNG Abs were entrapped within sol-gel monoliths containing various amounts of PEG having one of two molecular weights (0.4 or 10 kDa). The monoliths were gently crushed and IAP columns were prepared, in order to evaluate the activity of the entrapped Abs. In the present study the Abs did not exhibit significant differences in their ability to bind LNG in the presence or absence of various concentrations of 0.4- or 10-kDa PEG (Figure 1a). Empty sol-gels under all tested conditions did not differ significantly from each other. It may very well be that different Abs are affected to differing degrees by the presence of PEG and, being much smaller molecules than the acetyl-choline receptor, are less affected by differences between PEGs. 

SEM analysis of the structure of these sol-gels revealed typical branched silica nanoclusters, with nanometer-size pores. No marked structural difference was observed between the sol-gel samples and the silica skeleton, and the pore sizes in the presence and absence of 10% 0.4 kDa PEG appeared to be similar (Figure 1b, and c). Nevertheless, both textural and color differences have been observed between sol-gels containing PEG of differing molecular weights or in differing amounts (data not shown). In general, sample murkiness and fragility were increased by using larger amounts of PEG (up to 20%); and use of the high-MW 10-kDa PEG resulted in a milky white sol-gel in contrast to the transparent sol-gel obtained with the 0.4-kDa PEG. The above differences have also affected the column flow velocity. One explanation of the lack of structural difference in the presence and absence of PEG might be that the low-MW PEG (0.4-kDa) used in the present study had only a minor effect on the structure of the monolith, whereas only higher–MW (*i.e.*, ≥2,000) porogens affect the pore size and silica skeleton in a manner that can be clearly visualized by SEM. Support for this hypothesis can be found in the study of Sun *et al*., who demonstrated that monoliths formed in the presence of 0.4-kDa PEG differed markedly from those formed in the presence of higher-MW PEGs. It is possible that employment of other techniques, such as nitrogen-adsorption or atomic-force microscopy [24] might provide a more accurate determination of pore-size difference between the two formats.

### 2.2. Effects of Different Sol-Gel Formats on the Structure and Activity of Entrapped Abs

**Figure 2:**
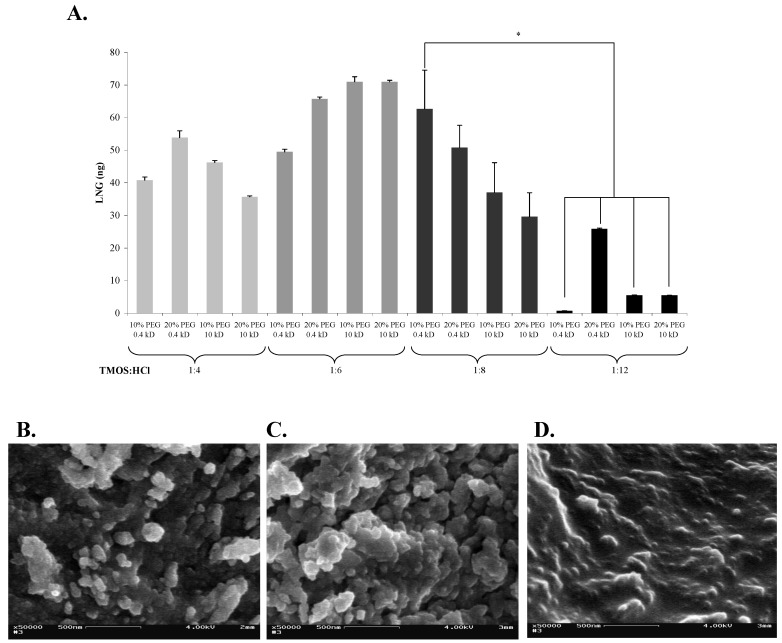
Effects of various sol-gel formats (characterized by differing TMOS:HCl ratios) on their structure and on the activity of entrapped Abs. (**A**) LNG (100 ng) was applied on 1-day-aged sol-gel columns prepared with 10 or 20% of 0.4-kDa or 10-kDa PEG, at four different TMOS:HCl ratios (1:4, 1:6, 1:8 and 1:12). Amounts of eluted LNG were determined by ELISA. Data are presented as net binding, *i.e.*, binding of LNG to Abs-doped columns minus the non-specific binding of LNG to 'empty' columns. Each vertical bar represents the mean ± S.E.M of n experiments, where: for TMOS:HCl = 1:8, n = 6; and for 1:4, 1:6, and 1:12, n = 2. An asterisk indicates significant differences at *p* < 0.05. (**B**), (**C**), and (**D**) are SEM images of the sol-gels prepared at various TMOS:HCl ratios: 1:4, 1:8, and 1:12, respectively, all containing 10% of 0.4-kDa PEG. Magnification, ×50,000; scale bar represents 500 nm.

Other parameters such as the silica (precursor)-to-water ratio, e.g., TMOS:H_2_O also affect the porosity of the sol-gel matrix. In general, an increase in the amount of H_2_O causes an increase in the hydrolysis rate, which, in turn, increases the number of active sites available for polymerization and results in formation of a matrix with smaller pores. Complete hydrolysis leads to formation of a matrix that consists of a dense SiO_2_ network. We have, therefore, tested the effects of different sol-gel formats on both the sol-gel structure and the activity of the entrapped Abs. We selected four TMOS:HCl ratios, and tested each with two PEG concentrations (10 and 20%), with PEG of two molecular weights (0.4 or 10 kDa). The highest Ab activity was obtained by using 1:6 or 1:8 formats (Figure 2a). The activity of the Abs in the 1:4 format was somewhat lower, but did not differ significantly from that in the 1:6 or 1:8 formats. The activities in all 1:12 formats were lower than those in any other format, and differed significantly from that exhibited by the 1:8 sol-gel that contained 10% of 0.4-kDa PEG. In general, substitution of 10-kDa PEG for 0.4-kDa PEG, or increasing the percentage from 10 to 20% in any of the sol-gel formats did not affect the activity of the entrapped Abs, except in the case of a 1:12 format in which addition of 20% of 0.4-kDa PEG resulted in a significant elevation of Abs activity. Non-specific binding values ranged from 3 to 29 ng. No correlation could be detected between a given format and an elevated non-specific binding. Examination of the binding capacity of the LNG Abs in the various formats indicated that the pore size affected the activity of entrapped Abs, and the activity in a high-density sol-gel (*i.e.*, at a TMOS:H_2_O ratio of 1:12), was, indeed, significantly lower than that in the other, more porous, sol-gels (Figure 2a), probably because the small pores constrained the mobility and conformation of the Abs. Similar results were obtained with a sol-gel-entrapped anti-dinitrophenyl Abs in which the activity of the entrapped Abs was significantly lower at a TMOS:H_2_O of 1:12 than at other ratios [23]. In light of the above results, the best sol-gel format for Abs entrapment was found to be a matrix with a TMOS:H_2_O ratio of 1:8, containing 10% of 0.4-kDa PEG. Examination of the structure of sol-gel matrices that were prepared at differing TMOS:H_2_O ratios by SEM indicated differences in pore sizes and matrix density with the 1:12 format being much denser than others, having smaller and fewer pores (Figure 2b, c, d)—supporting the above hypothesis. 

### 2.3. Effects of Different Monomers on the Structure of the Sol-gel and on the Activity of Entrapped Abs

**Figure 3:**
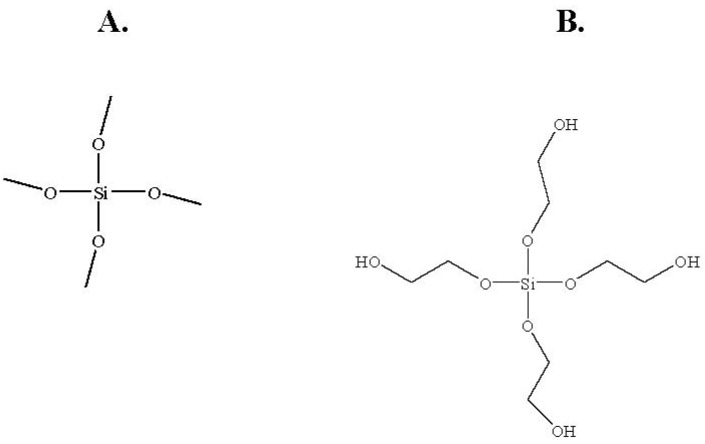
Structures of the sol-gel precursors: (**A**) TMOS; (**B**) THEOS.

**Figure 4:**
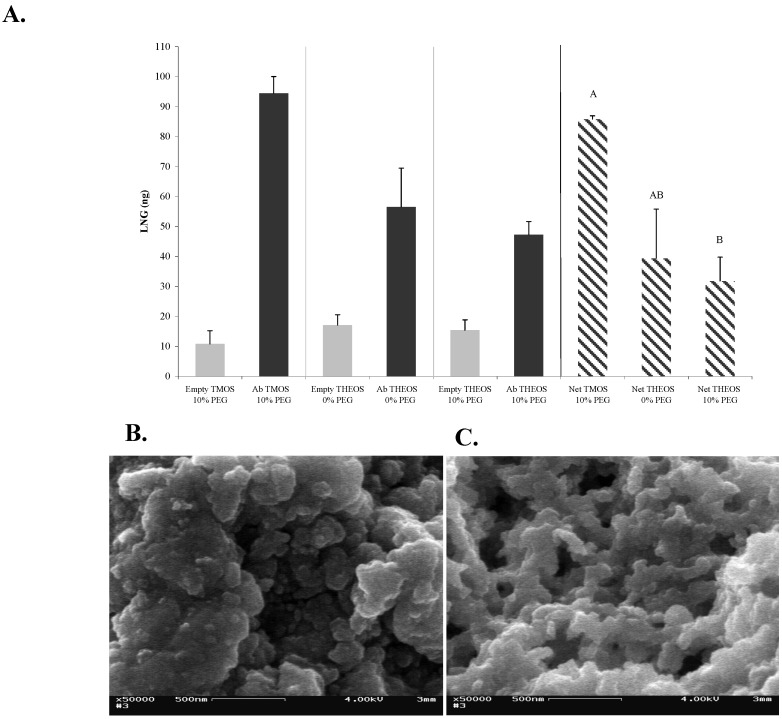
Effects of different monomers on the structure of the sol-gel and on the activity of entrapped Abs. (**A**) LNG (100 ng) was applied on 1-day-aged sol-gel columns prepared with TMOS or THEOS as precursor, at a precursor:HCl ratio of 1:8, with 10% of 0.4 kDa PEG. Amounts of eluted LNG were determined by ELISA. Each vertical bar represents the mean ± S.E.M of two experiments. Means with the same letter did not differ significantly at *p* < 0.05; (**B**) and (**C**) are SEM images of freshly prepared sol-gels based on 1:8 THEOS and 1:8 TMOS, respectively, containing 10% of 0.4-kDa PEG. All other details are as described in the legend to Figure 2. Magnification × 50,000; scale bar represents 500 nm.

Sol-gel-derived materials can be produced with a wide range of compositions. Nevertheless, TMOS and THEOS are the most frequently used precursors for immobilization of biomolecules, because of the simple procedures and mild hydrolysis conditions needed to generate the final matrix (for review see [25]). However, both TMOS and THEOS exhibit the serious disadvantage of liberating, during hydrolysis, significant amounts of alcohol that could cause protein denaturation upon entrapment. To overcome this problem, a number of sol-gel-derived materials have been designed to render the matrix more biocompatible with the entrapped biomolecules. For example, new biocompatible silane precursors and processing methods, based on glycerated silanes, have been reported. These groups reported remarkable improvements in activity of entrapped biomolecules, because of the sugar substituents. Previous studies performed in several laboratories, including ours, found that Abs activity within a TMOS matrix was very similar to that obtained in solution [26], therefore, it might be reasonable to consider that Abs are less sensitive than other biomolecules (e.g., receptors), to alcoholic by-products. Moreover, alkoxysilane precursors carry alkyl groups which add hydrophobic moieties to the monolith. Those moieties could affect the activity of entrapped biomolecules, such as Abs generated against highly hydrophobic antigens such as steroid hormones. The presence of such moieties would be a major disadvantage in the development of, for instance, sol-gel-based IAP methods, because the hydrophobic antigen might interact with the matrix, which would result in a high degree of non-specific binding. In order to determine whether a less hydrophobic precursor did, in fact, affect non-specific binding we compared the non-specific binding of LNG to a monolith generated from TMOS with that generated from THEOS, which is less hydrophobic [27]. In the present study we compared the activities of Abs entrapped in sol-gels generated from TMOS (Figure 3a) or THEOS (Figure 3b) as precursors. The results (Figure 4a) indicated that entrapment of Abs within a THEOS-based sol-gel matrix resulted in a lower activity, although a significant difference in performance was noticed only between TMOS-based sol-gels columns containing 10% of 0.4-kDa PEG and THEOS-based sol-gels containing 10% of 0.4-kDa PEG. The activity of the Abs in THEOS-based columns in the absence of PEG was lower than that in the TMOS-based columns, although the differences were not statistically significant. The results also indicated that in the case of LNG the non-specific binding did not depend on the precursor, but rather on the presence or absence of PEG. No differences in non-specific binding were found between TMOS- and THEOS-based sol-gels that contained 0.4-kDa PEG, but in the absence of PEG the non-specific binding was significantly higher in the THEOS-based than in the TMOS-based sol-gel (Figure 4a). Interestingly, addition of 10-kDa PEG instead of 0.4-kDa PEG, or use of a greater amount of 0.4-kDa PEG (20% instead of 10%) in the THEOS-based sol-gels prevented formation of a sol-gel monolith: the matrix obtained was slurry and did not enable Ab entrapment. Moreover, the polymerization time of a THEOS-based sol-gel was much shorter than that of a TMOS-based one (a few seconds), which forced us to change the sol-gel protocol as indicated in the Experimental Section. SEM images showed that the THEOS-based and TMOS-based sol-gels had a differing molecular structures (Figure 4b, c) and textures. The silica skeleton of the THEOS-based monolith was much larger than that of the TMOS-based and the pores were larger.

### 2.4. Effects of Aging on the Matrix Structure and on Abs Activity

**Figure 5:**
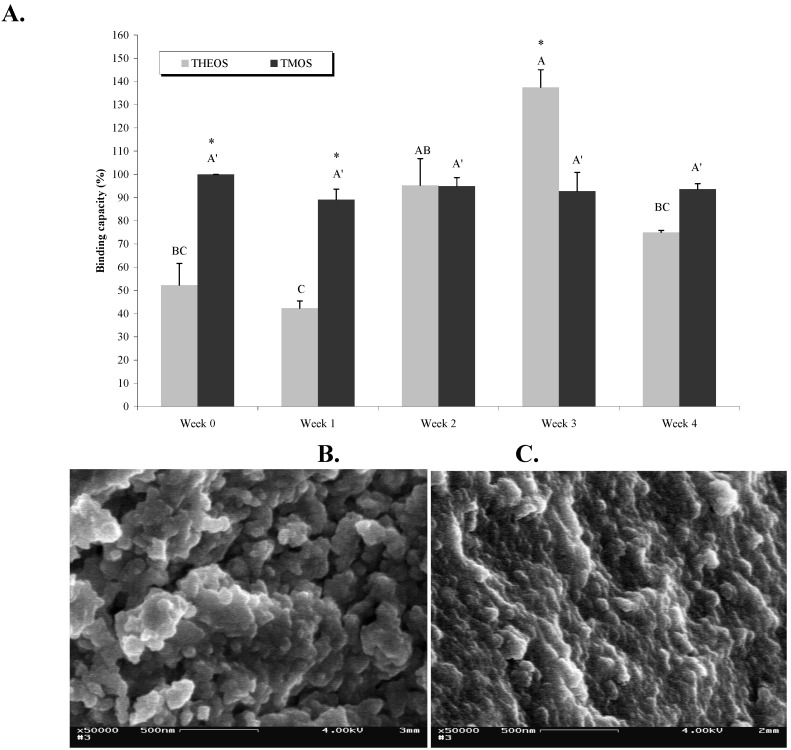
Effects of aging on matrix structure and on Abs activity. (**A**) LNG (100 ng) was loaded on columns containing sol-gels based on TMOS at a TMOS:HCl ratio of 1:8 containing 10% of 0.4-kDs PEG, or on THEOS at a THEOS:HCl ratio of 1:8 without PEG, as precursors. The columns were stored for periods of 1 day (Week 0) up to 4 weeks at 4 °C. Amounts of eluted LNG were determined by ELISA. Binding capacity represent the ratio (expressed as a percentage) between the amounts of LNG recovered from a TMOS or THEOS column at each time point and that from a freshly prepared TMOS sol-gel column (Week 0, designated as 100%). Each vertical bar represents the mean ± S.E.M of two experiments. Statistical analysis compared the binding capacity of doped TMOS- or THEOS-based sol-gel columns separately as a function of aging (indicated by letters) and of the binding capacity of TMOS *vs*. THEOS at each time point (indicated by an asterisk). Means with the same letter did not differ significantly at *p* < 0.05. Means marked with an asterisk (*) differed significantly at *p* < 0.05. (**B**) and (**C**), respectively, are SEM images of freshly prepared and 4-week-old TMOS-based sol-gels with a TMOS:HCl ratio of 1:8 containing 10% of 0.4-kDa PEG. Magnification, × 50,000; scale bar represents 500 nm.

During storage the sol-gel matrix undergoes an aging process that involves continuous condensation of the sol-gel, which thereby becomes denser, with smaller pores. In the present study we used TMOS and THEOS as precursors, and examined the effects of aging on both matrix structure and Abs activity. As can be seen in Figure 5a, whereas Abs entrapped in TMOS-based sol-gels retained the same activity level during four weeks of aging at 4 °C, the activity of Abs entrapped in THEOS-based sol-gels varied as a function of ‘aging’: it was low in the first two weeks but increased after two to three weeks of aging, and reached a significantly higher level than that of the TMOS-entrapped Abs. Aging for another week resulted in a significant decrease in activity (Figure 5a). The reason for the differing activity patterns of the two matrices might stem from the structural and textural differences, as revealed by SEM images (Figure 4b, c). The freshly prepared THEOS-based sol-gel seems to be more porous than the TMOS-based one and may, therefore, allow leaching of the Abs, which would lead to lower activity.

SEM images of aged TMOS-based sol-gel did, indeed, reflect the condensation process that resulted in a much denser matrix (Figure 5b and c). Apparently these density changes did not affect Abs activity in TMOS-based matrices. The activity of Abs entrapped in THEOS-based sol-gels dropped significantly after four weeks of aging. It is possible that the condensation rates of the two polymers differed, being faster in THEOS-based matrices. However, although it is reasonable to assume that long storage periods might eventually affect Abs activity because of pore shrinkage, the temporal behavior of this phenomenon is dependent, most likely, on the nature of the precursor, the hydrolysis conditions, and the additives in the polymer; our previous studies have shown that Abs entrapped in TMOS-based sol-gels were still active after up to nine months (Altstein, unpublished). In light of these results we may conclude that TMOS is more suitable than THEOS as a precursor for entrapment of LNG Abs. Examination of the condensation–polymerization process and of Abs activity over longer periods might provide a better understanding of the relationship between Abs activity and the matrix properties. This is particularly important for the development of IAP-based methods for the clean-up and concentration of hydrophobic agents from matrices other than water, e.g., milk, serum, soil, *etc*., where precursor selection might become an even more complex issue. Aged THEOS should still be considered, because it might help to lower the non-specific binding to the sol-gel matrix, of various hydrophobic components in the tested sample. 

### 2.5. Abs Distribution within the Gel

**Figure 6:**
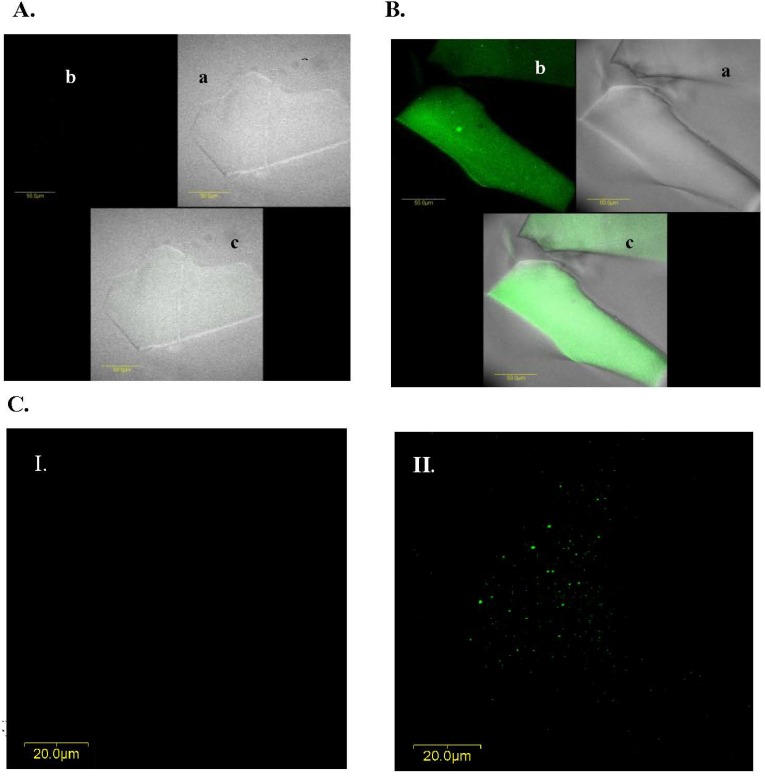
IgG distribution within a TMOS-based sol-gel prepared with a TMOS:HCl ratio of 1:8 containing 10% of 0.4-kDa PEG. **A**. Confocal microscopy image of ‘empty’ xerogel (*i.e*., sol-gel without IgGs). **B**. Images of xerogels containing FITC-labeled IgGs; xerogels were mounted on microscope slides and scanned with a fluorescent confocal microscope (**a**) phase image (without using laser beam at the tested wavelength); (**b**) fluorescence level of a doped sol-gel sample; and (**c**) superimposition of the phase and fluorescent images in (a) and (b). **C**. Confocal microscopy images of (I) empty, and (II) doped xerogels containing FITC-labeled IgGs and based on TMOS at a TMOS:HCl ration of 1:8 containing 10% of 0.4-kDa PEG. Images A and B were taken with a magnification of × 60; those in C were taken with a × 60 magnification and a × 2 zoom at a depth of 15 μm. The scale bars in (A) and (B) represent 50 μm; and in (C) represents 20 μm.

In addition to the effects of different precursors and additives on both Abs activity and matrix structure, we also examined the Abs distribution within the matrix: we purified IgG from the LNG antiserum, labeled them with FITC and entrapped them in a TMOS-based sol-gel containing 10% 0.4 kDa PEG. Confocal fluorescent microscopy analysis of ‘empty’ and doped sol-gels indicated that the entrapped Abs were distributed homogenously within the gel (Figure 6). Examination of the Abs distribution in other sol-gel formats, such as TMOS 1:4 or 1:12 containing 10% of 0.4-kDa PEG or THEOS 1:8 without PEG, revealed similar results (data not shown), indicating that the Abs distribution within the matrix was indeed homogenous and independent of the matrix composition. Previous studies that examined the same issue also revealed homogenous distribution of various biomolecules in the gel. For example, transmission electron microscopy of Pb-labeled lipases, and laser confocal microscopy of fluorescence-labeled lipases revealed that the enzyme was uniformly distributed in the matrix, and absorbance measurements of entrapped proteins such as cytochrome *c* have suggested that an entrapment procedure similar to that used in the present study led to homogenous dispersion of biomolecules on the macroscopic scale [28]. 

## 3. Experimental Section 

### 3.1. Polyclonal Ab 

Polyclonal anti-LNG antiserum was generated in rabbits by using a 3’-carboxymethyloxime (CMO) derivate of LNG, conjugated to bovine serum albumin (BSA) (Sigma, St. Louis, MI, USA) as an immunogen, as previously described. The polyclonal Abs were used throughout the study.

### 3.2. Purification of Anti-LNG Immunoglobulins (IgGs) from Anti-Serum 

Protein A agarose beads (Protein A agarose, binding capacity of 7 mg IgG/mL beads, Sigma, St. Louis, MI, USA) were packed in a Pasteur pipette at room temperature. The column was pre-washed with 8 mL of phosphate-buffered saline (PBS) (0.15 M NaCl in 50 mM sodium phosphate, pH 7.2), followed by an additional wash with 0.8 mL of 0.1 M citrate buffer, pH 3.5 (Citric Acid, Merck, Darmstadt, Germany) and washed again with 8 mL of PBS. A 1-mL aliquot of anti-LNG antiserum was diluted 1:10 in PBS and applied to the column. The eluate was collected and applied twice more, to ensure maximal binding. The column was washed with 20 mL of PBS and IgGs were eluted by four washes with 0.5 mL of citrate buffer, pH 3.5. The citrate buffer fractions were collected into tubes containing 0.5 mL of 1 M Tris (Trisma Base; Bio Lab, Jerusalem, Israel), pH 8.5, to obtain a final mixture pH of about 7. The eluates containing the purified IgGs were pooled concentrated by centrifugation in a Vivaspin 2 device (Sartorius, Goettingen, Germany), with a cut off at 30,000 kDa, for 10 min at 4,000 × g, and washed twice with PBS. The final volume of the IgG fraction was brought up to 1 mL by addition of PBS, and the IgGs were tested for their titer by enzyme linked immunosorbent assay (ELISA). The protein content was determined by a Bradford reaction in a Bio-Rad protein assay (Bio-Rad Laboratories, Munich, Germany), performed according to the vendor’s instructions. The protein content was 2 mg/mL.

### 3.3. Ab Labeling with Fluorescein Isothiocyanate (FITC) 

FITC (PIERCE, Rockford, IL, USA) was dissolved in DDW at a concentration of 1 mg/mL, and 10 μL of the solution were mixed thoroughly with 1 mL of purified IgG diluted 1:2 in 0.1 M carbonate buffer, pH 9.0. The reaction was incubated for 60 min in darkness at room temperature. Unbound FITC was removed by size exclusion with a Vivaspin 2 (Vivaspin 2, Sartorius); the reaction mixture was centrifuged for 10 min at 4,000 × g, followed by two washes with 1 mL of DDW. The final reaction volume was adjusted to 1 mL. All steps were carried out in darkness.

### 3.4. Ab Entrapment within Sol-Gel Matrices 

#### 3.4.1. TMOS-Based Sol-Gel Entrapment of Anti-LNG Anti-Serum

Entrapment involved a two-step procedure in which hydrolysis was followed by polymerization of tetramethylsilane (TMOS, Aldrich) as previously described [29]. Briefly, an acidic silica sol solution was obtained by mixing TMOS with 2.5 mM HCl in DDW at a TMOS:HCl molar ratio of 1:4, 1:6, 1:8, or 1:12, in the presence of 0–20% polyethylene glycol (PEG-400 or PEG-10,000, of MW 0.4 or 10 kDa, respectively, Merck, Darmstadt, Germany). The mixture was stirred for 1 min until a clear solution was obtained, and then sonicated for 30 min in an ELMA ultrasonicator bath (model T-460/H, 285 W, 2.75 L, Singen-Hohentwiel, Germany). The procedure was carried out in a well-ventilated fume hood. Anti-LNG antiserum (80 μL) was premixed with 50 mM 4-(2-hydroxyethyl)-1-piperazineethanesulfonic acid buffer (HEPES, 99.99%, Sigma) at pH 7.6, to a final volume of 0.5 mL and added to 0.5 mL of the prehydrolyzed TMOS mixture. Gels in which no antiserum was entrapped (i.e., ‘empty’ gels) were prepared by mixing the hydrolyzed TMOS with 0.5 mL of HEPES buffer, pH 7.6. The solution was mixed quickly for 5 s, and gelation occurred within 1–2 min. After 30 min, the gels (total volume of 1 mL) were washed with 2 mL of HEPES buffer at pH 7.6 and kept wet (with 2 mL of HEPES on top) at 4 °C pending use. In most of the experiments gels were used within 24 h of preparation, except in aging experiments, when they were kept at 4 °C for 1, 2, 3, or 4 weeks, and compared with a 24-h ‘freshly’ prepared gel (termed ‘week 0’ below). 

#### 3.4.2. THEOS-Based Sol-Gel Entrapment of Anti-LNG Anti-Serum

Entrapment involved a two-step procedure in which hydrolysis was followed by polymerization of tetrakis (2-hydroxyethyl)orthosilicate (THEOS, Sigma). Briefly, an acidic silica sol solution was obtained by mixing 1.39 mL of THEOS with 0.98 mL of 2.5 mM HCl in DDW (at a 1:8 molar ratio of THEOS:HCl), and 1.99 mL of HEPES pH 7.6 were added immediately to the mixture. The solution was stirred gently and a 920-μL aliquot from it was mixed with 80 μL of anti-LNG antiserum. In cases where the sol-gel was prepared with PEG, 100 μL of 0.4 or 10 kDa PEG were added to the THEOS/HCl mixture. ‘Empty’ gels (in which no antiserum was entrapped) were prepared by adding 80 μL of HEPES buffer, pH 7.6, instead of the antiserum, to 920 μL of the THEOS/HCl/HEPES mixture. The solution was mixed quickly for 5 s, and gelation occurred within 10 min. After 1 h, the gels (total volume of 1 mL) were washed with 2 mL of HEPES buffer at pH 7.6 and kept wet, with 2 mL of HEPES on top, at 4 °C pending use.

#### 3.4.3. TMOS or THEOS Based Xerogels

Xerogels (dry gels) were prepared similarly to the above. For confocal microscopy gels were prepared at a TMOS:HCl ratio of 1:8, containing 10% of 0.4-kDa PEG and 80 μL of anti-LNG FITC-labeled IgGs premixed with 50 mM HEPES buffer at pH 7.6 in a final volume of 0.5 mL were added to 0.5 mL of the prehydrolyzed TMOS mixture. For scanning electron microscopy (SEM) gels were prepared either from TMOS at 1:4, 1:8, or 1:12 ratios, with or without 10% of 0.4-kDa PEG, or from THEOS at 1:8 with 10% of 0.4-kDa PEG, as described above. No Abs were entrapped in any of the sol-gels that underwent SEM analysis. Gels were stored for 24 h at 4 °C, and vacuum dried to dryness. Xerogels were stored in sealed vials at 4 °C pending use, and were analyzed with a confocal microscope or SEM, as described below.

#### 3.4.4. Binding and Elution of LNG from Sol-Gel IAP Columns

Wet gels (TMOS- or THEOS-based) were thoroughly crushed and packed in 20 mL columns (Chromatography Columns 1.5 × 12 cm, Bio-Rad, Hercules, CA, USA). The sol-gel columns were washed with 50 mL of 0.15 M NaCl in 10 mM sodium phosphate, pH 7.2 (loading buffer) prior to sample application. For optimal binding, columns were kept under buffer throughout the experiment. A 100-ng aliquot of LNG standard in a volume of 1 mL of loading buffer was applied to ‘empty’ sol-gel columns (see below) or to columns doped with anti-LNG antiserum. Unbound LNG was removed by washing with 10 mL of DDW. Elution was performed with 10 mL of absolute ethanol (PESTI-S, Bio-Lab, Jerusalem, Israel). The eluted fraction underwent vacuum evaporation, to remove the eluting solvent, and was further concentrated by means of a solid-phase column as described below. Binding experiments were performed with pairs of sol-gel columns that comprised (A) an experimental column containing anti-LNG antiserum (for total binding) and (B) an empty control column without antiserum (for non-specific binding). Specific binding was defined as the difference between the total binding and the non-specific binding. All of the experiments described in the present paper were performed with a standard comprising a stock solution of LNG dissolved in ethanol at 1 mg/mL. 

#### 3.4.5. Solid-Phase Extraction (SPE): Sample Application and Elution

Oasis SPE columns (Waters, Milford, MA, USA) were preconditioned by two washes with 10 mL of absolute ethanol (PESTI-S, Bio-Lab, Jerusalem, Israel), followed by a single wash with 10 mL of 10% ethanol in DDW. Sol-gel-eluted samples were reconstituted in 1 mL of 10% ethanol in DDW. Samples were loaded on the columns, which were then washed with 10 mL of 10% ethanol in DDW. Elution was carried out with 1 mL of absolute ethanol. The samples underwent vacuum evaporation and were then dissolved in 1 mL of PBS. In order to eliminate any residual Abs activity that might be present in the eluate because of leakage from the sol-gel column, and that would interfere with the ELISA, samples were pretreated for 10 min at 100 °C prior to analysis. The LNG content was determined by competitive ELISA as described in section 3.5. (at a range of 10–0.0049 ng /50 μL). 

### 3.5. LNG Competitive ELISA 

The assay developed was an indirect competitive ELISA, in which tested compounds or LNG standards in solution competed with an antigen-protein conjugate immobilized on a 96-well microtiter plate, for binding to anti-LNG anti-serum. The assay served to determine the amounts of LNG eluted from the sol-gel IAP columns. Wells of microtiter plates (Nunc, F96 Maxisorp, Nunc Immuno Plate, Roskilde, Denmark) were coated with 100 μL of LNG-OVA conjugate (prepared as previously described), diluted 1:2,000 )0.34 μg per well) in 0.5 M carbonate buffer, pH 9.6. After an overnight incubation at 4 °C, the wells were washed three times with PBS containing 0.1% (v/v) Tween-20 (PBST), and 50 μL of test (unknown) samples or standard LNG (12 serial dilutions ranging from 10 to 0.0049 ng/well) were added to the wells, together with 50 μL of anti-LNG antiserum (diluted 1:5,000 in PBST containing 10% of ethanol). Plates were incubated overnight at 4 °C, and washed as above with PBST, and 100 μL of secondary Ab horseradish peroxidase (HRP) conjugated (antirabbit HRP conjugated, Sigma), diluted 1:30,000 in PBST, were added to the plates. The plates were incubated for 2 h at room temperature, rinsed with PBST, and tested for HRP activity by the addition of 100 μL of substrate solution 3,3’,5,5’-tetramethyl benzidine (TMB substrate chromagen, Dako, Glostrup, Denmark). The reaction was stopped after 10 min by the addition of 50 μL of 4 N sulfuric acid, and the absorbance was measured with a Labsystems Multiscan Multisoft ELISA reader at 450 nm. Content of LNG in unknown samples was determined by comparison with LNG calibration curves after transformation of the data by Origin software (OriginPro 7.5, Origin Lab, Northampton, MA, USA). Each sample was tested in duplicate at five dilutions. 

### 3.6. Microscopy 

#### 3.6.1. Scanning Electron Microscopy

Dry sol-gel (xerogels), prepared from either TMOS or THEOS monomers, in various formats, were placed on a carbon surface. The samples were coated with a thin layer of carbon in a customized coating machine (Carbon Coater, Polaron, Watford, UK) before the imaging process. Images of the various sol-gel formats were recorded with a Leo Gemini high-resolution SEM 982 camera (Leo Zeiss, Peabody MA). 

#### 3.6.2. Confocal Microscopy

Dry sol-gel powders (xerogels doped with FITC-labeled IgG or 'empty' controls) were placed on gelatin-coated microscope slides (Menzel Glaser, Braunschweig, Germany) and mounted with Moviol composed of 1 g polyvinyl alcohol 4-88 (Sigam) mixed with 4 mL of 0.015 M sodium phosphate buffer, pH 8.0, and 2 mL of glycerol (Ultra-Pure MD, Biomedicals, Mumbai, India). Samples were covered with a cover slip (18 mm, Menzel Glaser, Braunschweig, Germany) and kept for 24 h at 4 °C pending confocal microscopic examination. All microscopic examinations and image acquisitions were carried out with an Olympus IX-81 laser confocal microscope (FV 500, Olympus Optical Co., Tokyo, Japan) equipped with a 488-nm argon-ion laser and a PlanApo × 60 objective. Transmitted-light images were obtained by using Nomarski differential interference contrast (DIC) microscopy. For FITC detection, samples were excited by 488-nm light and the emission was collected through a BA 505IF filter. 

### 3.7. Statistics 

Differences between mean values were determined by Tukey-Kramer one-way ANOVA at *p* < 0.05. 

## 4. Conclusions 

In conclusion, the present study provided a broad insight into sol-gel-entrapped Abs activity within various matrices and tried to correlate their activity with the matrix structure. It provides a better understanding of Abs entrapment in various matrices, and helps in the practical application of the technology to IAP and other sol-gel-based immunochemical methods. The findings clearly indicate that different biomolecules behave differently under different entrapment conditions, and that Abs may be more promiscuous than entrapped enzymes or receptors. Although sol-gel-derived biocomposites have been shown to be useful in various analytical applications, many unresolved questions regarding parameters still require further study, and optimization for individual biomolecules. Because the use of sol-gel biocomposites for various applications is a new field of research, it is envisioned that accumulation of more data will lead to faster progress in its applications, and that that, in turn, will lead to the emergence of new and improved devices for applications in environmental, forensic, agricultural and clinical studies. 
